# Aberrant Epigenetic Silencing Is Triggered by a Transient Reduction in Gene Expression

**DOI:** 10.1371/journal.pone.0004832

**Published:** 2009-03-12

**Authors:** Jon A. Oyer, Adrian Chu, Sukhmani Brar, Mitchell S. Turker

**Affiliations:** 1 Department of Molecular and Medical Genetics, Oregon Health & Science University, Portland, Oregon, United States of America; 2 Center for Research on Occupational and Environmental Toxicology (CROET), Oregon Health & Science University, Portland, Oregon, United States of America,; Oregon State University, United States of America

## Abstract

**Background:**

Aberrant epigenetic silencing plays a major role in cancer formation by inactivating tumor suppressor genes. While the endpoints of aberrant silencing are known, i.e., promoter region DNA methylation and altered histone modifications, the triggers of silencing are not known. We used the tet-off system to test the hypothesis that a transient reduction in gene expression will sensitize a promoter to undergo epigenetic silencing.

**Methodology/Principal Findings:**

The tet responsive promoter (*P_TRE_*) was used to drive expression of the selectable human *HPRT* cDNA in independent transfectants of an *Hprt* deficient mouse cell line. In this system, high basal *HPRT* expression is greatly reduced when doxycycline (Dox) is added to the culture medium. Exposure of the *P_TRE_-HPRT* transfectants to Dox induced *HPRT* deficient clones in a time dependent manner. A molecular analysis demonstrated promoter region DNA methylation, loss of histone modifications associated with expression (i.e., H3 lysine 9 and 14 acetylation and lysine 4 methylation), and acquisition of the repressive histone modification H3 lysine 9 methylation. These changes, which are consistent with aberrant epigenetic silencing, were not present in the Dox-treated cultures, with the exception of reduced H3 lysine 14 acetylation. Silenced alleles readily reactivated spontaneously or after treatment of cells with inhibitors of histone deacetylation and/or DNA methylation, but re-silencing of reactivated alleles did not require a new round of Dox exposure. Inhibition of histone deacetylation inhibited both the induction of silencing and re-silencing, whereas inhibition of DNA methylation had no such effect.

**Conclusions/Significance:**

This study demonstrates that a transient reduction in gene expression triggers a pathway for aberrant silencing in mammalian cells and identifies histone deacetylation as a critical early step in this process. DNA methylation, in contrast, is a secondary step in the silencing pathway under study. A model to explain these observations is offered.

## Introduction

Aberrant epigenetic silencing is a common and significant mechanism in cancer development and progression [1]. Like mutational events, aberrant silencing frequently inactivates tumor suppressor genes in both sporadic tumors and human cancer cell lines [2]. Unlike mutations, however, silencing is a stepwise process [3], [4] with potential for reversal [5]. These observations have led to research to identify the molecular changes that accompany silencing. Such changes include promoter region DNA methylation, histone deacetylation, histone methylation at specific residues (*e.g.* H3K9, H3K27), and densely packed nucleosomes that create a closed chromatin structure [6]. However, a caveat is that these changes are most often documented at stably silenced alleles that were under continuous selective pressure within the tumor microenvironment for maintenance of the silenced state. Therefore, reported epigenetic modifications represent an ultimate endpoint and do not reveal how silencing initiates, nor do they reveal the order of epigenetic modifications that occur during the transition from active expression to stable silencing. Such information is required to create strategies to prevent the initiation or progression of aberrant epigenetic silencing.

Many models designed to examine initiation of silencing track normal epigenetic changes during development at imprinted genes [7] or during X chromosome inactivation [8], but developmentally programmed silencing may progress differently than aberrant silencing occurring in cancer. Promoter DNA methylation is the most common modification associated with epigenetic silencing, and has previously been thought to play a causal role [9], but evidence is accumulating to suggest DNA methylation as a late step in the silencing process. For example, DNA methylation occurs after histone modifications for silenced, stably integrated transgenes [10]. A similar progression of epigenetic modifications occurs for silencing of the endogenous tumor suppressor gene *RASSF1A* [11]. Previous studies in our laboratory showed that silencing of an integrated *Aprt* transgene allows the spread of DNA methylation into a promoter region, which stabilizes the silenced transcriptional state [4]. Although DNA methylation has been the most common modification associated with cancer-related silencing, examples of epigenetic silencing occurring independent of DNA methylation show it is not an absolute requirement [12]–[14]. Collectively these data suggest that DNA methylation primarily functions to maintain and stabilize the silenced state and that other epigenetic processes are required to initiate silencing.

If DNA methylation is neither a required nor an initiating step for aberrant silencing, how is this process triggered? Recent studies suggest reduced expression as one possibility. For example, in ovarian cancer loss of the *GATA6* transcription factor results in reduced expression and subsequent epigenetic silencing of the downstream target *Disabled-2* [15]. Also, inhibition of ERα (estrogen receptor α) signaling in breast cancer cell lines reduces expression and induces silencing of the downstream target gene, *PR* (progesterone receptor) [16]. These are two instances that involve loss of transcriptional activators, but evidence also exists that reducing expression by inappropriate recruitment of transcriptional repressors can lead to silencing. An inherited mutation in the *DAPK1* promoter apparently causes B-cell chronic lymphocytic leukemia by increased localization of a transcriptional repressor that reduces expression and correlates with silencing [17]. In addition to altered signaling pathways, some environmental changes accompanying tumor progression also reduce gene expression, which could initiate silencing. For example, hypoxia. a common feature of tumor microenvironments, represses expression of tumor suppressor genes (e.g., *E-CAD*, *BRCA1*, *MLH1*, and *RUNX*) [18]–[21] frequently silenced in cancer [2], [22], [23].

In the current study we have developed a system to directly test the hypothesis that a transient reduction in gene expression can sensitize a promoter to undergo epigenetic silencing. The results demonstrate that this principle is correct. Additionally, we find that induction of silencing is dependent on histone deacetylase activity, but does not require DNA methylation.

## Results

### A system to study transient reductions in gene expression

**Figure 1:**
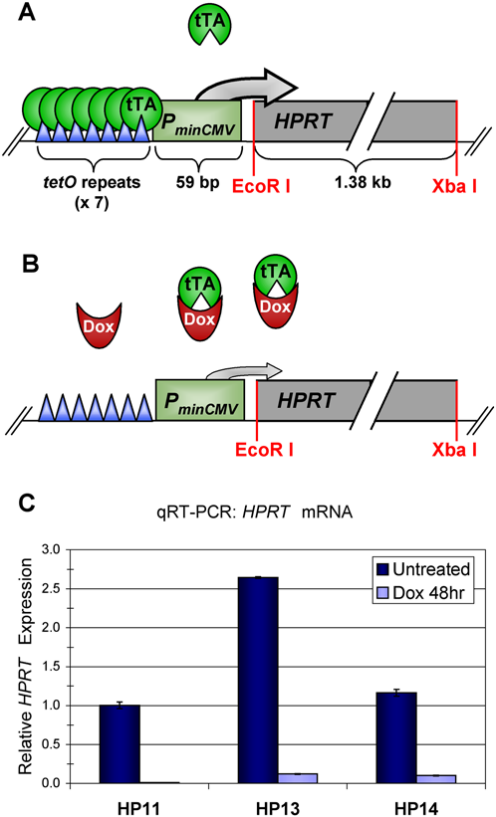
Treatment with doxycycline (Dox) reduces *P_TRE_-HPRT* expression. (A) The tet-off system was used to express *HPRT*. The 1.38 kb *HPRT* cDNA was cloned into the 5′ EcoR I and 3′ Xba I restriction sites downstream of the minimal CMV promoter (P_minCMV_). In the absence of Dox, tTA binds to the seven 19 bp tet operator (tetO) sequence repeats in the promoter and activates high expression of *HPRT*. (B) Adding Dox reduces *HPRT* expression by direct binding of tTA, though minimal expression levels remain. (C) *HPRT* mRNA levels are significantly reduced within 48 hours of growth in media containing 1 µg/ml Dox. *HPRT* expression was measured by quantitative RT-PCR (qRT-PCR) and normalized to *Gapdh* expression levels. Each bar represents the average of triplicate reactions with error bars indicating minimum and maximum fold change.

To test the hypothesis that a transient reduction in gene expression can initiate epigenetic silencing, we used the tet-off system [24] to control transcription levels of human *HPRT* cDNA in the mouse Dif-6 cell line, which lacks expression of endogenous *Hprt* [25]. In this system the tet-Transcriptional Activator (tTA) localizes to the tet-responsive promoter (*P_TRE_*) and promotes *HPRT* expression (Fig. 1A). Adding the tetracycline analog doxycycline (Dox) to the growth medium reduces *HPRT* expression by directly binding tTA and inhibiting its localization to the promoter (Fig. 1B). Three stable transfectants, HP11, HP13, and HP14, expressing high levels of *HPRT* were established. After 48 hours growth in Dox medium, *HPRT* expression was reduced by more than 90% relative to untreated controls, with the HP11 cell line exhibiting the strongest Dox response (Fig. 1C). Although *HPRT* expression is significantly reduced, cell cultures growing in Dox media remain sensitive to selection against *HPRT* (Fig. S1) and can grow under conditions that require *HPRT* expression (data not shown). A concentration of 1 µg/ml Dox showed a maximum effect on expression without causing toxicity (data not shown) and was used in all subsequent Dox treatments.

### A transient reduction in gene expression induces phenotypic gene inactivation

**Figure 2:**
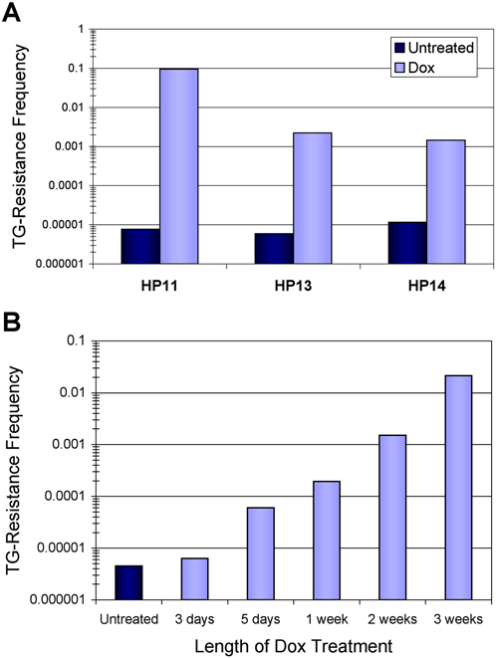
Dox exposure induces *P_TRE_-HPRT* inactivation. (A) Reducing expression of *P_TRE_-HPRT* by growing cells for 1 week in medium containing 1 µg/ml Dox increased the frequency of gene inactivation, as measured by TG-resistance. TG-resistance was measured by washing out Dox, selecting cells with 5 µg/ml TG, and counting surviving colonies after 2 weeks of continuous selection. During Dox treatment or the equivalent period without treatment, cells were maintained in medium containing puromycin and G418 to maintain the *P_TRE_-HPRT* and tTA constructs respectively, but without azaserine/hypoxanthine (AzHx) selection. Only cells that express *HPRT* can grow in AzHx selection. (B) TG-resistance frequencies increased as a function of time HP13 cells were exposed to Dox before starting TG selection. HP13 cells were continuously cultured in 1 µg/ml Dox for 3 weeks, and TG-resistance was measured at different points during the Dox treatment (3, 5, 7, 14, and 21 days). A parallel control culture was maintained in medium containing puromycin and G418 without Dox, and TG-resistance was measured after 21 days (Untreated).

Following removal of Dox from the culture medium the tTA protein can again bind to *P_TRE_* and restore *HPRT* expression. However, our hypothesis predicts that during the period of reduced expression a fraction of alleles will become epigenetically silenced and thus will be unable to restore *HPRT* expression upon removal of Dox. To test this hypothesis, cells were grown in Dox media for a week to reduce expression and allow adequate time for HPRT protein turnover before removing Dox and selecting for *HPRT* deficient cells with the purine analog 6-thioguanine (TG). The fraction of surviving TG-resistant cells reflects the gene inactivation frequency for *P_TRE_-HPRT*. Dox exposure was found to induce TG-resistant cells for all three cell lines, at frequencies ranging from 1.4×10^−3^ to 9.4×10^−2^, which were several orders of magnitude higher than untreated cultures (Fig. 2A). Moreover, *P_TRE_-HPRT* inactivation frequency increased with longer durations of initial Dox exposure (Fig. 2B). These results demonstrate that transient reductions in gene expression correlate with greatly increased frequencies of *P_TRE_-HPRT* inactivation.

### Epigenetic modifications consistent with silencing characterize inactivated *P_TRE_-HPRT* alleles

**Figure 3:**
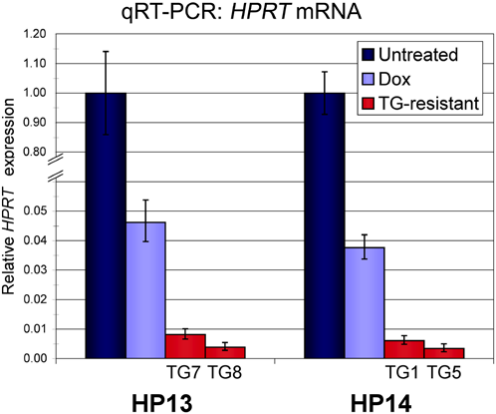
*P_TRE_-HPRT* inactivation correlates with reduced mRNA levels. Dox-induced TG-resistant clones were isolated and expanded. *HPRT* mRNA levels in TG-resistant clones from the HP13 (TG7 and TG8) and HP14 (TG1 and TG5) parental cells are lower than both active expression levels (Untreated) and reduced expression levels after exposure to Dox for one week (Dox). *HPRT* mRNA was measured by qRT-PCR and normalized to *Gapdh* expression levels. Each bar represents the average of triplicate reactions with error bars indicating minimum and maximum fold change.

**Figure 4:**
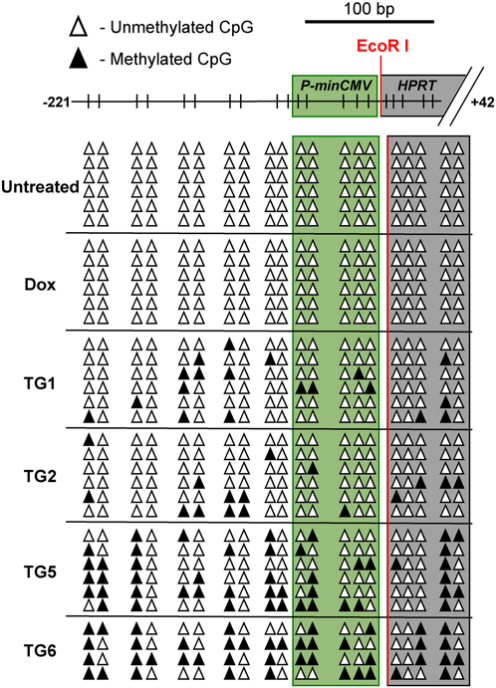
*P_TRE_-HPRT* inactivation correlates with increased promoter DNA methylation. Allelic methylation patterns for parental HP14 cells expressing high levels of *HPRT* (Untreated), reduced levels of *HPRT* after an 1 week Dox treatment (Dox), and HP14-derived TG-resistant clones (TG1, TG2, TG5, and TG6). Bisulfite sequencing identified methylated (closed triangles) and unmethylated (open triangles) CpG sites within individual alleles. Schematic of the promoter shows approximate positioning of CpG sites (vertical bars) within the minimal CMV promoter (green shaded box) and the 5′ region (∼42 bp) of the *HPRT* cDNA sequence (grey shaded box). The start of the *HPRT* cDNA sequence, EcoR I restriction site, has been designated base position +1.

Individual TG-resistant clones were characterized to identify molecular changes correlating with *P_TRE_-HPRT* inactivation. TG-resistant clones were isolated from the HP13 and HP14 parental lines following one week of Dox treatment. *HPRT* mRNA levels in all TG-resistant clones were substantially lower than those observed in the Dox treated parental cells (Fig. 3). DNA methylation at the *P_TRE_-HPRT* promoters of HP14-derived TG-resistant cells was measured via bisulfite sequencing (Fig. 4). As expected, all CpG sites within the *P_CMVmin_*, *tetO* repeats, and nearby regions were unmethylated in actively expressing HP14 cells. Moreover, these sites remained unmethylated in the same cells grown in the presence of Dox for one week. In contrast, all TG-resistant clones analyzed exhibited DNA methylation in the promoter region, though the density of DNA methylation varied (Fig. 4). TG1 and TG2 both exhibited low levels of DNA methylation and contained some alleles without any methylated CpG sites in the minimal CMV promoter (*P_minCMV_*). In contrast, TG5 and TG6 exhibited substantially more DNA methylation within the promoter, including the core *P_minCMV_* region. The other two TG-resistant lines, TG3 and TG4, contained intermediate to high levels of DNA methylation within the promoter relative to the other cell lines (Fig. S2).

**Figure 5:**
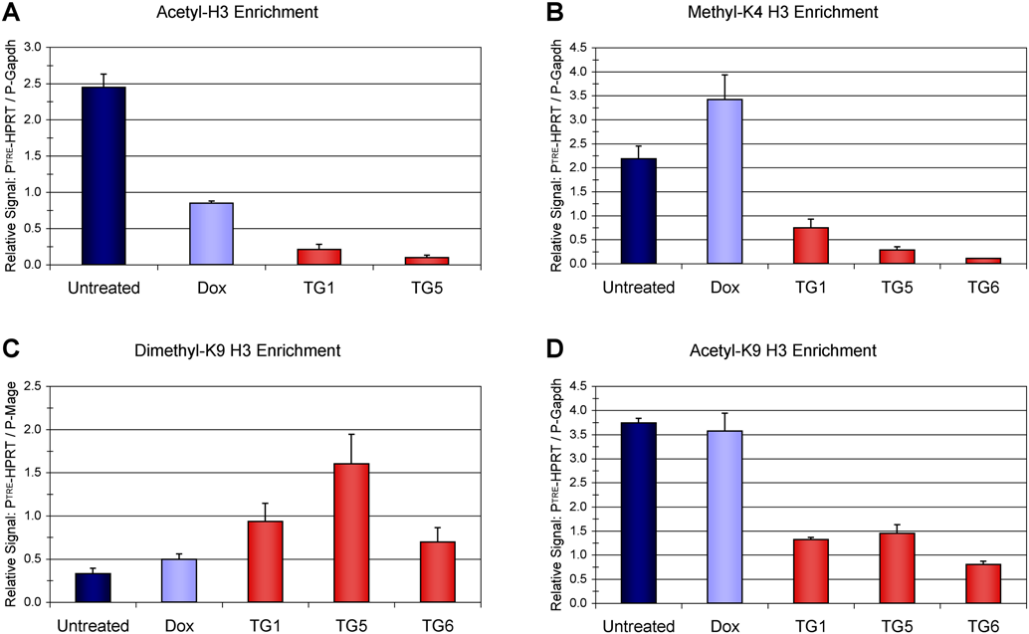
*P_TRE_-HPRT* inactivation correlates with repressive histone modifications. ChIP analysis measuring histone H3 modifications at the *P_TRE_-HPRT* promoter in HP14 cells expressing high levels of *HPRT* (Untreated), reduced levels of *HPRT* after 1 week Dox treatment (Dox), and HP14-derived TG-resistant cell lines (TG1, TG5, and TG6). (A) ChIP analysis measuring acetylated histone H3 using a polyclonal antibody raised against a peptide corresponding to acetyl-K9 and acetyl-K14. (B) ChIP analysis measuring methylation at lysine 4 of histone H3 (methyl-K4 H3). The antibody used for immunoprecipitation recognizes all three forms of methylation at K4, mono-, di-, and tri-methyl. (C) ChIP analysis measuring the repressive modification of dimethylation at lysine 9 of histone H3 (di-methyl-K9 H3). (D) ChIP analysis measuring acetylation at lysine 9 of histone H3 (acetyl-K9 H3). Immunoprecipitated DNA levels were quantified by qRT-PCR. Specific signal was calculated by measuring fold change between pull down and input at the *Hprt* promoter (*P_TRE_-HPRT*), *Gapdh* promoter (*P-Gapdh*), and *Mage-a* promoter (*P-Mage-a*). For activating modifications, levels at *P_TRE_-HPRT* are displayed relative to the *Gapdh* promoter; for the repressive modification, dimethyl-K9 H3, results are displayed relative to the *Mage* promoter. Error bars indicate the SD from triplicate reactions.

The absence of dense promoter DNA methylation in some clones suggested additional mechanisms were contributing to *P_TRE_-HPRT* inactivation. Chromatin immunoprecipitation (ChIP) analysis was used to measure specific histone modifications associated with either active transcription (methyl-K4, acetyl-K9, and acetyl-K14 of histone H3) or silenced transcription (dimethyl-K9 of histone H3) at the *P_TRE_-HPRT* promoter (Fig. 5). As expected, cells expressing high levels of *HPRT* have a histone modification pattern at the promoter consistent with active transcription. Specifically, the actively transcribed *P_TRE_–HPRT* promoter was associated with high levels of H3 acetylation (Fig. 5A) and methylation at lysine 4 (methyl-K4 H3) (Fig. 5B) relative to modification levels measured at the active *Gapdh* promoter (*P-Gapdh*). The repressive modification dimethyl-K9 H3 was low in the *HPRT* expressing cells (Fig. 5C), measured relative to the silenced *Mage-a* locus (*P-Mage*) [26], [27]. Reducing *HPRT* expression by treating cells with Dox did not reduce levels of methyl-K4 H3 (Fig. 5B) or significantly change the levels of dimethyl-K9 H3 (Fig. 5C) at the *P_TRE_-HPRT* promoter. However, H3 acetylation decreased significantly after reducing *HPRT* expression by Dox treatment (Fig. 5A). The antibody used for the acetyl-H3 ChIP recognizes both acetyl-K9 H3 and acetyl-K14 H3 [28]. To probe this decrease further, an additional ChIP was conducted with antibody directed specifically against acetyl-K9 H3. In this case, no decrease was observed after the one-week exposure to Dox (Fig. 5D). Therefore, reducing expression by Dox treatment caused loss of acetyl-K14 H3 without decreasing other modifications associated with transcriptional activity, i.e., methyl-K4 H3 or acetyl-K9 H3. Dox treatment had no effect on histone modifications measured at the control promoters (*P-Gapdh* and *P-Mage*) used for normalization.

In contrast to the observations for histone modifications in the presence of Dox, ChIP analysis for the Dox-independent, TG resistant clones revealed markedly reduced levels of methyl-K4 H3 and acetyl-K9 H3. Increased dimethyl-K9 H3 was also observed at the *P_TRE_-HPRT* promoter in the TG-resistant cells, ranging from a 2-fold increase in TG6 to a nearly 5-fold increase in TG5 (Fig. 5C). These results demonstrate that the transition from reduced expression to gene inactivation is associated with a shift from activating to repressive histone modifications consistent with epigenetic silencing.

### Silenced *P_TRE_-HPRT* alleles are reactivated by inhibiting histone deacetylases or DNA methylation

**Figure 6:**
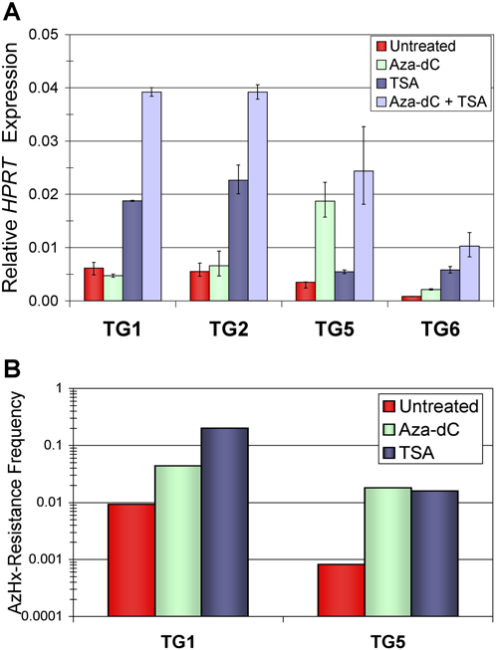
Silenced *P_TRE_-HPRT* alleles are reactivated by inhibiting histone deacetylation or DNA methylation. (A) Inhibition of DNA methylation and histone deacetylation increased *HPRT* mRNA levels. HP14-derived TG-resistant cell lines (TG1, TG2, TG5, and TG6) were treated with 300 nM 5-aza-dC (Aza-dC), inhibiting histone deacetylation with 100 nM trichostatin A (TSA), or a combination of the 300 nM 5-aza-dC and 100 nM TSA treatments (Aza-dC+TSA). Cells were treated with inhibitors overnight (∼16 hours), and RNA was harvested 24 hours later. The units shown along the Y-axis are relative to those measured in the untreated parental HP14 cells (see Figure 3). *HPRT* expression was measured by qRT-PCR and normalized to *Gapdh* expression levels. Each bar represents the average of duplicate reactions with error bars indicating minimum and maximum fold change. (B) TG-resistant cell lines were capable of reactivating *P_TRE_-HPRT* expression. Cells were plated with azaserine/hypoxanthine (AzHx) selection, which requires HPRT enzyme activity for cell survival, to isolate and measure the number of cells that reactivated *HPRT* expression. Before plating and selection, cells were treated overnight with 300 nM 5-aza-dC (Aza-dC), 100 nM TSA (TSA), or vehicle control (untreated) and allowed to recover for 24 hours. Frequencies represent the fraction of cell colonies surviving after two weeks of continuous AzHx selection.

One of the hallmarks of epigenetic silencing is reversibility. To confirm definitively that the induced *P_TRE_–HPRT* inactivation was due to silencing, we measured the effects of inhibiting histone deacetylation and/or DNA methylation on gene reactivation in the TG-resistant cells. First, changes in *HPRT* mRNA levels were measured for the TG-resistant cells after inhibiting histone deacetylation with trichostatin A (TSA) treatment or inhibiting DNA methylation with 5-aza-2′-deoxycytidine (5-aza-dC) (Fig. 6A and Fig. S3). The different TG-resistant clones had varied responses to histone deacetylase (HDAC) inhibition ranging from an approximately 3-fold increase in *HPRT* mRNA (TG1 and TG2) to no response (TG5). Inhibiting DNA methylation gave a nearly reciprocal result with the TG5 cell line showing the largest 5-aza-dC induction of *HPRT* mRNA, an approximately 5-fold increase, and little response in the TG1 and TG2 clones, which exhibited the strongest response after HDAC inhibition. Combining inhibition of histone deacetylases and DNA methylation by treating the cells with 5-aza-dC and TSA simultaneously resulted in synergistic induction of *HPRT* expression for every TG-resistant cell lines except for TG5, which exhibited at best an additive effect (Fig. 6A and Fig. S3).

Next we determined if the silenced alleles could phenotypically reactivate by selecting for revertant cells in media requiring *HPRT* expression for survival (azaserine / hypoxanthine or AzHx). Two TG-resistant cell lines, TG1and TG5, spontaneously gave rise to AzHx-resistant colonies at frequencies of 9.3×10^−3^ and 8.2×10^−4^, respectively (Fig. 6B). TSA and 5-aza-dC treatments were used to determine if inhibiting histone deacetylases or DNA methylation, respectively, would induce phenotypic reactivants similar to their effects on induced reactivation at the RNA level (Fig. 6B and Fig. S3). Phenotypic reactivants were induced, though the results did not mimic precisely those obtained by measuring *HPRT* mRNA levels. For example, TSA treatment increased the frequency of phenotypic reactivation of the TG5 cell line despite the apparent lack of induction when measuring *HPRT* mRNA one day after TSA treatment. While these discrepancies reveal differences between the two assays, the combined results clearly demonstrate that TG-resistance was due to epigenetic mechanisms.

### Reactivated alleles exhibit memory of transcriptional silencing

**Figure 7:**
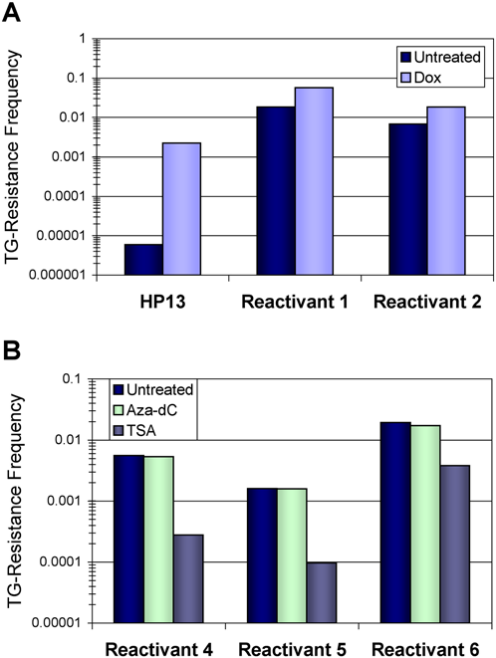
Reactivation of silenced *P_TRE_-HPRT* alleles is unstable. (A) *P_TRE_-HPRT* inactivation frequencies for HP13-derived clones with epigenetically silenced and then reactivated *HPRT* expression (Reactivant 1 and 2) were measured after one week without Dox exposure (Untreated) or after one week Dox treatment (Dox). The silencing frequency for the parental HP13 cell line (HP13) is shown for comparison. (B) *P_TRE_-HPRT* inactivation frequencies for HP14-derived clones with epigenetically silenced and then reactivated *HPRT* expression (Reactivants 4–6) were measured after overnight treatment with 300 nM 5-aza-dC (Aza-dC), 100 nM TSA (TSA), or vehicle control (Untreated) before selecting against *HPRT* activity with TG. Frequencies represent the fraction of cell colonies surviving after two weeks of TG selection.

Several laboratories have reported that 5-aza-dC reactivated promoters exhibit rapid re-silencing [29], [30]; however these experiments could not use selection to maintain expression. Our system allowed continuous selection to ensure maintenance of the reactivated promoter state by growing the cells in AzHx medium, which requires HPRT enzyme activity for cell survival. We therefore asked whether promoter reactivation stabilized under selective conditions or alternatively whether the reactivated promoters retained a memory of silencing, as defined by high frequency re-silencing. Although selection ensures *HPRT* expression, the absolute expression levels were variable ranging from 14% to 90% of *HPRT* expression in the parental cells (Fig. S4). Two clones, reactivants 1 and 2, were isolated from TG-resistant HP13 cells that had spontaneously reactivated *HPRT* expression and grew well in AzHx medium. Spontaneous and Dox-induced silencing frequencies were determined for both clones (Fig. 7A). These reactivant cell lines spontaneously re-silenced at high frequencies, 1.8×10^−2^ and 6.8×10^−3^, relative to the initial HP13 silencing frequency of 4.5×10^−6^, with Dox treatment only inducing an approximately 3-fold increase in silencing frequencies.

Three reactivant clones from the HP14 TG1 cell line (reactivants 4–6) were examined and also showed that Dox treatment was not required for high frequency *P_TRE_-HPRT* re-silencing (Fig. 7B). The spontaneous silencing frequency for parental HP14 cells was less than 6×10^−6^ (Fig. 2A), but all reactivant cell lines had spontaneous silencing frequencies (10^−3^ to 10^−2^) equal to or higher than that induced by the week-long Dox treatment (∼10^−3^). Knowing that the silenced state was reversible by inhibiting DNA methylation or histone deacetylation, we examined whether either of these events were required for re-silencing. After inhibiting DNA methylation with 5-aza-dC, the re-silencing frequencies were essentially unchanged relative to the high spontaneous frequencies. In contrast, HDAC inhibition by treatment with TSA reduced the re-silencing frequencies from 5- to 20-fold. In total, these results showed that the reactivated cells no longer required a period of Dox-mediated transcriptional reduction to silence expression and suggested that re-silencing was dependent on histone deacetylation, but not DNA methylation.

### Initiation of silencing is dependent on histone deacetylase activity but not DNA methylation

**Figure 8:**
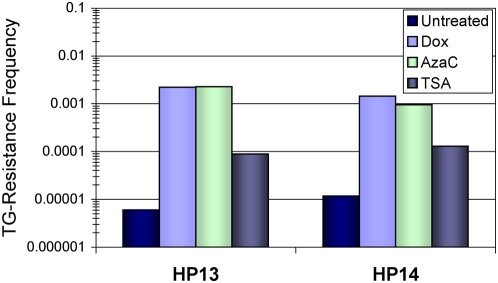
Histone deacetylase inhibition prevents Dox-induced *P_TRE_-HPRT* silencing. *P_TRE_-HPRT* inactivation frequencies for HP13 and HP14 cells were measured after treatment exposure to 1 µg/ml Dox for one week (Dox), exposure to Dox for one week plus 300 nM 5-aza-dC for the last 16 hours (Aza-dC), exposure to Dox for one week plus 100 nM TSA for the last 16 hours (TSA), or no treatments (Untreated). Frequencies represent the fraction of cell colonies surviving after two weeks of continuous TG selection.

After demonstrating that HDAC inhibition reduced re-silencing of reactivated alleles, we tested whether inhibiting HDACs or DNA methylation would affect initial silencing induced by Dox treatment. Induced silencing frequencies were measured again for the HP13 and HP14 parental *HPRT* expressing cell lines, with the modification of adding TSA or 5-aza-dC for the last 16 hours the cells were in Dox media. Inhibiting DNA methylation did not significantly affect the Dox-induced silencing frequency, but HDAC inhibition drastically reduced the silencing frequency (Fig. 8). These results show that HDAC activity is an early requirement for silencing induced by decreased transcription in our model, but DNA methylation is not required.

## Discussion

Aberrant epigenetic silencing is a significant mechanism of tumor suppressor gene inactivation, but how this process initiates in mammalian cells is poorly understood. We used the tet-off system to test the hypothesis that a transient and reversible reduction in gene expression could sensitize a promoter to undergo silencing. This hypothesis was based on observations showing reduced gene expression correlates with subsequent tumor suppressor gene silencing (see Introduction) and results from our laboratory showing transcriptional silencing allowed DNA methylation to spread into a promoter region [4]. Moreover, some tumor suppressor genes that are frequently silenced in cancer are also repressed by specific environmental exposures. For example, the tumor microenvironment causes hypoxia, which represses the *E-Cadherin* [18], *BRCA1* [19], and *MLH1* [20] tumor suppressor genes. All are epigenetically silenced in one or more cancer types [2], which suggests a relation between transcriptional repression and silencing in cancer. Here we report results from an experimental system that allowed us to demonstrate that a reduction in gene expression can trigger epigenetic silencing.

Dox treatment reduces expression in the tet-off system by preventing association of the tTA activator protein with the promoter, but reduced expression is not equivalent to epigenetic silencing. For example, the Dox-treated cultures remained sensitive to TG while silenced clones are TG-resistant (Fig. S1). However, a small fraction of cells exposed to Dox exhibited *HPRT* levels that are reduced further, which provided TG-resistance, and the fraction of cells increases with longer durations of Dox exposure. The induced TG-resistance was also relatively stable because it did not require continued exposure to Dox. These observations demonstrate that the reduced expression in the presence of Dox sensitized some alleles to undergo epigenetic silencing, but was insufficient to confer TG-resistance by itself. All evidence obtained in these experiments supported the conclusion that Dox-induced TG-resistance was due to epigenetic silencing as opposed to mutational events. The best evidence was the ability of TG-resistant cells to reactivate expression and restore functional HPRT activity, which was evident by growth of the cells in AzHx media. Besides the nine different TG-resistant clones described in this paper, we examined an additional fifteen TG-resistant clones induced by Dox treatment. At least one characteristic of epigenetic silencing (i.e., TSA or 5-aza-dC induction of *HPRT* mRNA or reactivant cell clones) was measured in each of these TG-resistant clones. In total, all twenty-four of the examined Dox-induced clones were shown to have silenced *P_TRE_-HPRT* alleles. Additionally, the silencing frequencies induced by Dox treatment were orders of magnitude higher than that expected for *HPRT* inactivating mutations (<10^−6^), and a previous study that characterized base-pair substitutions in the Dif-6 cells showed they do not have a mutator phenotype [31]. Dox treatment has also been used extensively in cell culture without having displayed mutagenic properties.

High-level promoter expression in the tet-off system occurs via localization of the tTA protein and activity of its VP16 activation domain; this domain promotes expression through recruitment of TBP, TFIIB, and the SAGA complex [32]. Then reduced expression during Dox treatment likely results from losing recruitment of these factors. The resultant disruption in recruitment of the SAGA complex and its associated histone acetyltransferases may therefore cause the concurrent decrease in acetyl-K14 H3. In contrast, acetyl-K9 H3 did not decrease when gene expression was reduced by Dox treatment, which demonstrates that the acetylation state of K9 and K14 of H3 may be regulated independently. Previous studies have also observed acetylation at K9 H3 can remain high despite decreased gene expression levels [33]. Levels of the repressive histone modification dimethyl-K9 H3 remained relatively low during reduced expression in the presence of Dox, which is not surprising considering the continuing presence of acetyl-K9 H3 should prevent the addition of methyl groups at K9 H3. Hypoxic conditions have been reported to increase dimethyl-K9 H3 upon repression of the mouse *Mlh1* [34] and human *RUNX3* promoters [21]. Increased dimethyl-K9 H3 has also been reported to result from nickel exposure [35], which can induce silencing of a *gpt* transgene in hamster cells [36]. While reduced expression alone did not induce methylation of K9 H3 in our system, increased levels of dimethyl-K9 H3 were observed after alleles transitioned to the silenced state identified by TG-resistance.

Results provided by our experiments help establish specific distinctions between the states of transcriptional repression and epigenetic silencing. In our system epigenetic silencing was defined as *HPRT* expression that was reduced to levels that allowed growth in TG selection. Therefore, the most evident difference was that clones with silenced alleles were TG-resistant while cells growing in Dox remained sensitive to TG selection (Fig. S1). The molecular basis of this phenotypic difference was demonstrated by showing TG-resistant cells had lower levels of *HPRT* mRNA than cells treated with Dox (Fig. 3) and molecular changes associated with epigenetic silencing (Figs. 4, 5, and S2). While the reduced expression after Dox treatment correlated with a loss of acetyl-K14 H3 at the *P_TRE_-HPRT* promoter, TG-resistance and epigenetic silencing correlated with additional molecular changes including DNA methylation, reduced methyl-K4 H3, loss of acetyl-K9 H3, and increased dimethyl-K9 H3 at the *P_TRE_-HPRT* promoter. Although increased DNA methylation was one of the molecular changes observed at silenced promoters in our system, DNA methylation was not required for the initiation of silencing because 5-aza-dC treatment had no effect on the frequency of silenced clones induced by Dox treatment. Evidence that the 5-aza-dC treatment used here was sufficient to inhibit DNA methylation was provided with experiments showing 5-aza-dC treatment induced reactivation of silenced *P_TRE_-HPRT* promoters that were hypermethylated (Fig. 6 and S3). Additionally, bisulfite sequencing analysis showed *HPRT* silencing in the TG1 and TG2 cell lines did not require high levels of DNA methylation (Fig. 4). In contrast to inhibition of DNA methylation, inhibiting HDAC activity prevented most, but not all, of the Dox-dependent increase in *HPRT* silencing. This observation suggests the presence of two populations of silenced alleles at the end of the Dox treatment. One population would be silenced alleles that are readily reactivated by TSA treatment, and the second population would be alleles that are more stably silenced and fail to restore *HPRT* expression after TSA treatment. Presumably, the second population would have acquired additional repressive epigenetic modifications that cooperate with histone deacetylation to stabilize the silenced state.

**Figure 9:**
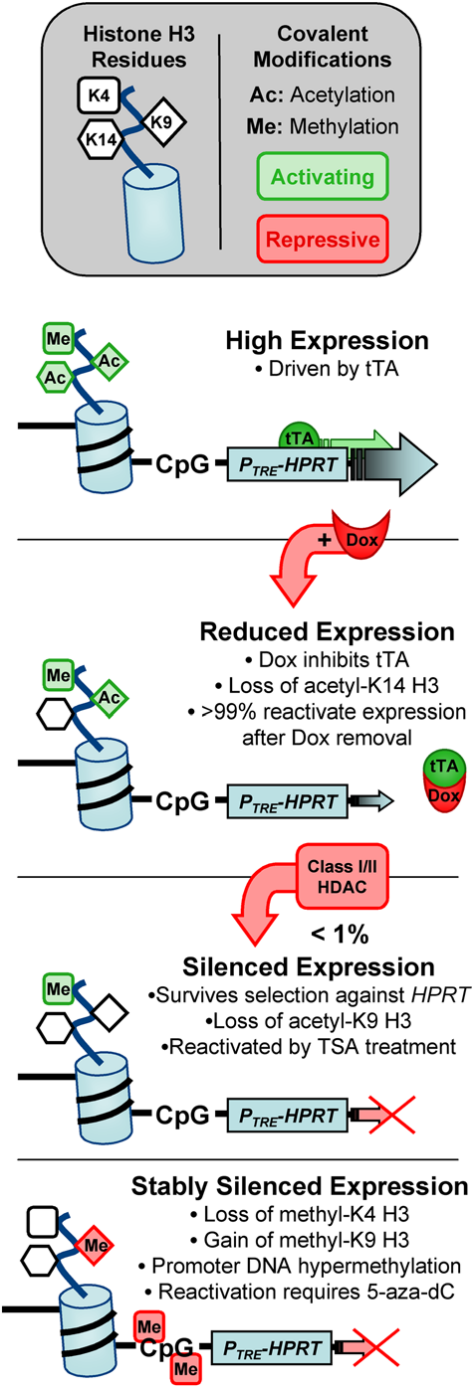
A model for induced silencing via reduced gene expression. (A) The VP16 activation domain promotes high levels of expression. DNA in the promoter region DNA is unmethylated and histone H3 is enriched for activating modifications (methyl-K4, acetyl-K9, and acetyl-K14). (B) Adding Dox reduces expression levels and acetylation at K14 of histone H3. (C) Reduced expression sensitizes alleles to undergo silencing; silenced alleles become unable to restore expression after Dox removal. The transition to silencing correlates with a further reduction in detectable mRNA and hypoacetylation at K9 H3 and is inhibited by TSA treatment (Class I/II HDAC inhibitor). (D) Additional epigenetic changes (loss of methylation at K4-H3, methylation at K9-H3, and DNA methylation) occur with continued TG selection against *HPRT* expression, as the silenced state stabilizes.

A speculative model (Fig. 9) to explain the results obtained herein is that promoters with high transcriptional activity are resistant to silencing and are characterized by epigenetic modifications commonly associated with active expression (Fig. 9A). After transcriptional activity decreases at the promoter, acetyl-K14 H3 levels are reduced, and the promoter is more susceptible to epigenetic silencing (Fig. 9B). Although decreased acetyl-K14 H3 alone is not sufficient to induce epigenetic silencing, loss of this modification could decrease protection of the promoter from epigenetic silencing. Similarly, histone H3 acetylation has been shown to establish a protective boundary against spreading of DNA methylation [10]. The transition from reduced expression to epigenetic silencing initiates with histone deacetylation based on the observations that acetyl-K9 H3 levels were low at silenced *P_TRE_-HPRT* promoters and inhibiting class I and II HDACs reduced the frequency of epigenetic silencing (Fig. 9C). Initially the silenced alleles are unstable and can be reactivated by TSA treatment, but as additional epigenetic modifications occur the silenced state stabilizes and is resistant to TSA treatment alone (Fig. 9D). We propose DNA methylation as a late step in epigenetic silencing because 5-aza-dC treatment did not affect the initiation of silencing. Although loss of methyl-K4 H3 is also shown as a secondary step, our results are not inconsistent with this loss being an early step in epigenetic silencing similar to loss of acetyl-K9 H3. While future experiments are required to test this model directly, aspects of it are consistent with prior observations. One is that silencing is a multistep process in which DNA methylation occurs downstream of silencing initiation [4]. This conclusion is supported by multiple observations of DNA methylation occurring after histone modification [4], [10], [11], [37] and examples of DNA methylation-independent silencing [12]–[14].

A current focus in cancer treatment is reactivating silenced tumor suppressor genes in malignant cells through the use of pharmacological agents [5]. Although inhibiting DNA methylation and histone deacetylation usually reactivates expression of silenced alleles [38], [39], such renewed expression is often unstable and quickly re-silences at a high frequency, possibly as a consequence of retention of some repressive histone modifications [29], [30]. Although temporary reactivation of tumor suppressor genes may be sufficient to induce anti-tumor effects, re-silencing would ensure that these effects are short-lived. Thus, it would be helpful to know if high frequency re-silencing reflects a lack of prolonged expression, or alternatively if silenced and reactivated alleles have a persistent memory of the silenced state. To distinguish these possibilities, we isolated subclones from cells with silenced *P_TRE_-HPRT* that spontaneously reactivated expression and used selection for *HPRT* to maintain the reactivated state for at least one month (∼50 cell divisions). Despite the prolonged time of reactivated expression, the absolute level of expression is not always restored to the original level (Fig. S4), and the reactivated *P_TRE_-HPRT* alleles still re-silence at a high frequency. Additionally, re-silencing did not require the Dox-mediated reduction in expression that was required for the initial silencing event. Thus, the memory of silencing was clearly persistent and likely reflects retention of epigenetic modifications. The inhibition of re-silencing with TSA suggests similarities with the initiation of silencing, which was also inhibited with TSA treatment.

We propose that the *P_TRE_-HPRT* system presented in this study represents a valid model for initiation and progression of aberrant silencing in cancer because silenced *P_TRE_-HPRT* alleles display the hallmarks of tumor suppressor gene silencing (promoter region DNA methylation, histone hypoacetylation, loss of methyl-K4 H3, and gain of methyl-K9 H3). In other words, we believe that the principle of reduced expression as a trigger for silencing will apply to *bona fide* mammalian promoters. Although our system utilized a non-mammalian promoter, endogenous levels of enzymes that control histone modifications and DNA methylation were responsible for the transition from repression to silencing. This is a unique and significant difference between our experimental system and previous systems that induced silencing by direct recruitment of repressive protein domains [40] or direct establishment of DNA methylation [41], [42] at promoters. Hence, our system has the potential to detect multiple independent pathways of epigenetic silencing, which could be cell type specific. For example, histone modifications and DNA methylation are both observed at silenced promoters in colon cancer cells, whereas some of the same promoters only exhibit histone modifications when silenced in prostate cancer cells [14].

In summary, we used the tet-off system to provide a clear demonstration that reduced transcriptional potential can sensitize a promoter to undergo epigenetic silencing. Consistent with prior work, the results demonstrate that silencing is a multistep process in which promoter region DNA methylation is secondary to altered histone modification. We propose that these results are applicable to tumor suppressor promoters that are repressible by internal or external environmental exposures and that the model we created will be useful for identifying molecular determinants of aberrant silencing in mammalian cells.

## Materials and Methods

### Tet-Off Constructs

The Tet-Off system has been described previously [24]. The pTet-Off plasmid (Clontech) expresses the neomycin (*Neo*) resistance gene (*Neo^r^*) and tTA, a fusion protein composed of the amino-terminus of the tetracycline repressor and the activation domain of the VP16 protein. The 1.38 kb *HPRT* full-length cDNA sequence (Accesion # NM_000194.1) was isolated by EcoRI and XbaI digestion of the TrueClone *HPRT* cDNA expression vector (Origene, catalog #TC120047). pTRE-tight-HPRT was created by directionally cloning the *HPRT* fragment into the EcoRI and XbaI restriction sites within the pTRE-Tight (Clontech) multiple cloning site.

### Cell Culture

Dif-6 cells were cultured in Dulbecco's modified Eagle's medium (Hyclone) supplemented with 5% fetal bovine serum (Hyclone) and 5% Serum Plus (SABC Biosciences). 5×10^6^ Dif-6 cells were transfected by electroporation [25] with 4 µg of pTet-Off plasmid (Clontech) that expressed the tTA activating protein and selected for linked *Neo^r^* with 500 µg/ml of G418. A transfectant showing high tTA expression was selected for a second transfection with 10 µg of *P_TRE_-HPRT* and 2 µg of a separate plasmid containing a bacterial puromycin (*pur*) resistance gene. Stable transfectants expressing functional *HPRT* were selected with media containing 10 µg/ml azaserine (Sigma) and 10 µg/ml hypoxanthine (Sigma) (AzHx medium). Selection for the *pur* gene was with 1.5 µg/ml puromycin (Invitrogen). Individual clones were expanded and screened for physical linkage between *pur* and *P_TRE_-HPRT* by identifying clones with low frequency *P_TRE_-HPRT* inactivation (via TG selection) while retaining resistance to puromycin. Comparing the *P_TRE_* promoter signal to the *Gapdh* promoter signal from genomic DNA samples by quantitative-PCR measured *P_TRE_-HPRT* copy number in the cell lines. HP11 and HP14 contained single copies and HP13 contained two copies of *P_TRE_-HPRT*. These parental cells were routinely cultured in AzHx, G418, and puromycin to retain expression all constructs and expression of *P_TRE_-HPRT*. Doxycycline hyclate (Dox) (Sigma) was added to DMEM at a concentration of 1 µg/ml for silencing experiments. Dox medium also contained G418 and puromycin to retain the tTA and *P_TRE_-HPRT* constructs, respectively. These drugs were also used during TG selection to retain both constructs in clones with silenced alleles. Cell exposed to Dox were not exposed to AzHx, unless indicated.

### RNA Preparation and Analysis

Total RNA was isolated from cell cultures with the RNeasy Mini Kit (Qiagen) according to manufacturer's instructions. Total RNA samples were converted to cDNA using Quantitect Reverse Transcription Kit (Qiagen) with removal of genomic DNA contamination. 100 ng cDNA was used as input in subsequent quantitative-PCR analysis for either *HPRT* (TaqMan assay Hs99999909_m1, Applied Biosystems) or *Gapdh* (Mouse TaqMan Endogenous Control, Applied Biosystems) with iQ Supermix (Bio-Rad) and a Bio-Rad iCycler. *HPRT* Results were normalized in relation to *Gapdh* mRNA levels and displayed relative to an arbitrary value.

### Silencing and Reactivation Cell Cloning Assays

To measure *P_TRE_-HPRT* inactivation or reactivation, cells were plated into 100 mm plates at densities ranging from 1×10^4^ to 1×10^5^ cells per plate. The next day the medium was removed, cells were rinsed with DMEM, and TG or AzHx selective medium was to select against or for *HPRT* expression, respectively. Cells were cultured for approximately two weeks in the appropriate selective media before staining live colonies with crystal violet solution. To estimate cloning efficiencies, additional cells were plated under identical conditions as selective plates but at lower densities, 250 to 1000 cells per plate and without selection for or against *HPRT* expression. Silencing or reactivation frequencies were calculated by dividing the number of clones growing under selection by the effective number of cells plated (as determined with the cloning efficiency plates).

### Drug Treatments

Cells were treated with media containing 100 nM TSA (Wako) overnight (∼16 hours) to inhibit histone deacetylation. Cells were treated with media containing 300 nM 5-aza-dC (Sigma) overnight (∼16 hours) to inhibit DNA methylation. For *HPRT* mRNA analysis, cells were allow to recover 24 hours in DMEM after drug treatment (TSA or 5-aza-dC) before harvesting for RNA purification.

### DNA Methylation Bisulfite Sequencing Assay

Genomic DNA was isolated from cell cultures using DNAzol (Molecular Research Center) according to the manufacturer's instructions. For each treatment, 4 µg of genomic DNA was digested with Bsr I, and modified in a solution of 6.24 M urea, 4 M sodium bisulfite, and 10 mM hydroquinone as described previously [4]. PCR amplification of modified DNA, cloning of PCR products, and sequence analysis were also described elsewhere [42], with the following exceptions. The primers used in the initial PCR reaction were the TRE-NaBis-S sense primer 5′-GTA TTT ATT AGG GTT ATT GTT TTA TGA G-3′ and the HPRT NaBis-A antisense primer 5′-CAA AAT AAA TCA AAA TCA TAA CCT AAT TC-3′. 1 µl of the PCR product was used as input in the subsequent semi-nested PCR reaction using the TRE-NaBis-NS primer 5′-GTA TTT AGA AAA ATA AAT AAA TAG GGG TTT-3′ and HPRT-NaBis-A for amplification. PCR products were cloned using Strataclone PCR cloning kit (Stratagene). Sequencing analysis showed all cytosine bases not present in the CpG dinucleotide context were converted to thymine indicating complete bisulfite modification of the genomic template occurred.

### Chromatin Immunoprecipitation

ChIP assays were carried out using EZ ChIP chromatin immunoprecipitation kit (Millipore) with the following specific details or modifications. Proteins were cross-linked to DNA in 5×10^7^ cells by adding formaldehyde to a final concentration of 1% and incubating for 10 minutes at room temperature. The cross-linking reaction was stopped by addition of glycine to a final concentration of 125 mM and incubating for 5 minutes at room temperature. Cells were rinsed with cold PBS containing complete protease inhibitor cocktail (Roche) and resuspended in SDS lysis buffer. Lysates were sonicated using a Branson 450 microtip sonicator to shear DNA into 100–1000 bp fragments. Protein-DNA complexes were immunoprecipitated using antibodies to acetyl-K9/K14 H3 (06-599, Millipore), acetyl-K9 H3 (07-352, Millipore), mono/di/trimethyl-K4 H3 (05-791, Millipore), and dimethyl-K9 H3 (ab1220, Abcam). 5 µl of each specific antibody was added to lysates from ∼1×10^6^ cells and incubated overnight at 4°C. Immunocomplexes were isolated by incubating for 3 hours at 4°C with a 3∶1 mixture of Protein A and Protein G conjugated magnetic Dynabeads (Invitrogen) that had been blocked with salmon sperm DNA and BSA. Beads were washed once with each of the following: low salt buffer, high salt buffer, LiCl buffer, and 1× TE. Immunocomplexes were eluted by incubating beads at 65°C for 15 minutes in 200 µl elution buffer (50 mM Tris-HCl, 10 mM EDTA, 1% SDS), and the cross-links were reversed by incubating at 65°C overnight. After incubation with 0.2 µg/ml RNase A at 37°C for 2 hours and 0.2 µg/ml Proteinase K at 55°C for 2 hours, DNA was purified using QiaQuick PCR purification kit (Qiagen). Quantitative PCR using an Icycler and iQ SYBR Green Supermix(Bio-Rad) was used to analyze the immunoprecipitated DNA. The *P_TRE_-HPRT* promoter was amplified using the 5′-AAC GTA TGT CGA GGT AGG CGT GTA-3′ sense promoter and the 5′-ATC TCC TTC ATC ACA TCT CGA G-3′ antisense promoter. The active *Gapdh* promoter was amplified using the 5′-TTG AGC TAG GAC TGG ATA AGC AGG-3′ sense promoter and the 5′-AAG AAG ATG CGG CCG TCT CTG GAA-3′ antisense promoter. The silenced *Mage-a* promoter was amplified using the 5′-GTT CTA GTG TCC ATA TTG GTG-3′ sense promoter and the 5′-AAC TGG CAC AGC ATG GAG AC-3′ antisense promoter. The specific signal from each immunoprecipitation relative to signal from input was calculated for the three promoters, *P_TRE_-HPRT*, *Gapdh*, and *Mage*. For activating modifications, levels at *P_TRE_-HPRT* are displayed relative to the *Gapdh* promoter; for the repressive modification, dimethyl-K9 H3, results are displayed relative to the *Mage* promoter.

## Supporting Information

Figure S1 Dox treated cells remain sensitive to HPRT selection. Growth curves of the HP13 (red) and HP14 (blue) cells that had been treated with Dox for one week to reduce HPRT expression and allow for protein turnover. After a week, the cells were maintained in Dox in the presence (dashed lines) or absence (solid lines) of 5 µg/ml TG (solid lines).
(0.10 MB TIF)

Figure S2 PTRE-HPRT inactivation correlates with increased promoter DNA methylation. Expanded allelic methylation patterns for parental HP14 cells expressing high levels of HPRT (Untreated), reduced levels of HPRT after a 1 week Dox treatment (Dox), and HP14-derived TG-resistant clones (TG1–TG6). Bisulfite sequencing identified methylated (closed triangles) and unmethylated (open triangles) CpG sites within individual alleles. Schematic of the promoter shows approximate positioning of CpG sites (vertical bars) within the minimal CMV promoter (green shaded box) and the 5′ region (∼112 bp) of the HPRT cDNA sequence (grey shaded box). The start of the HPRT cDNA sequence, EcoR I restriction site (red vertical bar), has been designated base position +1. The HPRT start codon is marked by the orange vertical bar.
(3.99 MB TIF)

Figure S3 Silenced PTRE-HPRT alleles are reactivated by inhibiting histone deacetylation or DNA methylation. (A) Inhibition of DNA methylation and histone deacetylation increased HPRT mRNA levels. HP14-derived TG-resistant cell lines (TG3 and TG4) were treated with 300 nM 5-aza-dC (Aza-dC), inhibiting histone deacetylation with 100 nM trichostatin A (TSA), or a combination of the 300 nM 5-aza-dC and 100 nM TSA treatments (Aza-dC+TSA). Cells were treated with inhibitors overnight (∼16 hours), and RNA was harvested 24 hours later. The units shown along the Y-axis are relative to those measured in the untreated parental HP14 cells (see Figure 3). HPRT expression was measured by qRT-PCR and normalized to Gapdh expression levels. Each bar represents the average of duplicate reactions with error bars indicating minimum and maximum fold change. (B) TG-resistant cell lines were capable of stably reactivating PTRE-HPRT expression as shown by reversion assay. Cells were plated with azaserine/hypoxanthine (AzHx) selection, which requires HPRT enzyme activity for cell survival, to isolate and measure the number of cells that stably reactivated HPRT expression. Before plating and selection, cells were treated overnight with 300 nM 5-aza-dC (Aza-dC), 100 nM TSA (TSA), both 300 nM 5-aza-dC and 100 nM TSA (Aza-dC+TSA), or vehicle control (Untreated) and allowed to recover for 24 hours. Frequencies represent the fraction of cell colonies surviving after two weeks of continuous AzHx selection.
(0.26 MB TIF)

Figure S4 Reactivant cell lines have increased HPRT expression levels. HPRT mRNA levels were measured in reactivant cell lines (Reactivants A, B, C, and D) and displayed relative to the expression level in the initial HP13 parental cell line (Untreated). Also shown for comparison are HPRT expression levels in the TG-resistant cell line before reactivation (TG), and the parental line after treatment with Dox for one week (Dox). HPRT expression was measured by qRT-PCR and normalized to Gapdh expression levels. Each bar represents the average of duplicate reactions with error bars indicating minimum and maximum fold change.
(0.18 MB TIF)
