# Supplementary figures and images for: Aberrant Epigenetic Silencing Is Triggered by a Transient Reduction in Gene Expression
Source: PLoS One. 2009 Mar 12;4(3):e4832. doi: 10.1371/journal.pone.0004832 (PMC2654015; doi:10.1371/journal.pone.0004832)

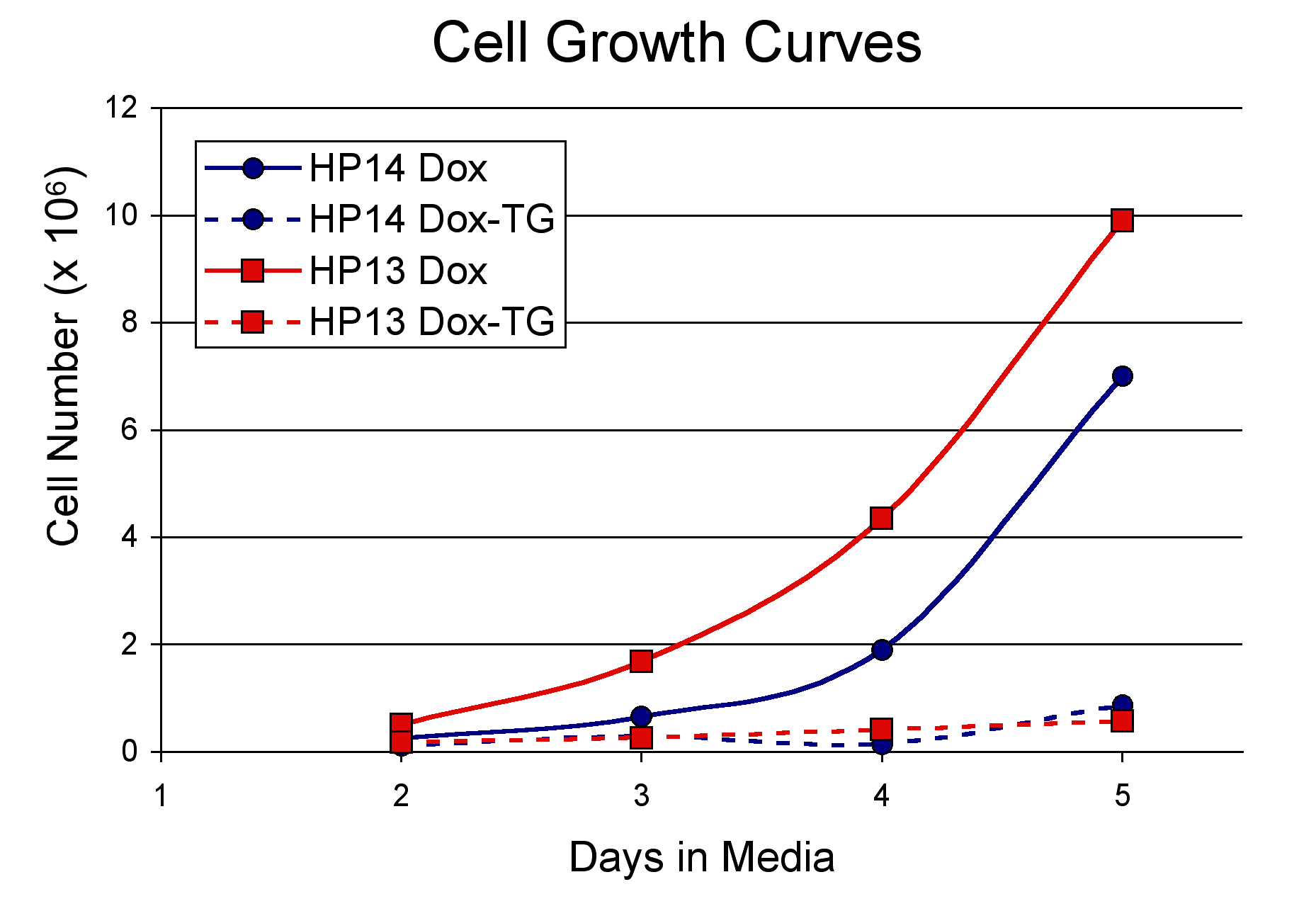

Supplement: Figure S1 — Dox treated cells remain sensitive to HPRT selection. Growth curves of the HP13 (red) and HP14 (blue) cells that had been treated with Dox for one week to reduce HPRT expression and allow for protein turnover. After a week, the cells were maintained in Dox in the presence (dashed lines) or absence (solid lines) of 5 µg/ml TG (solid lines). (0.10 MB TIF) [file pone.0004832.s001.tif]

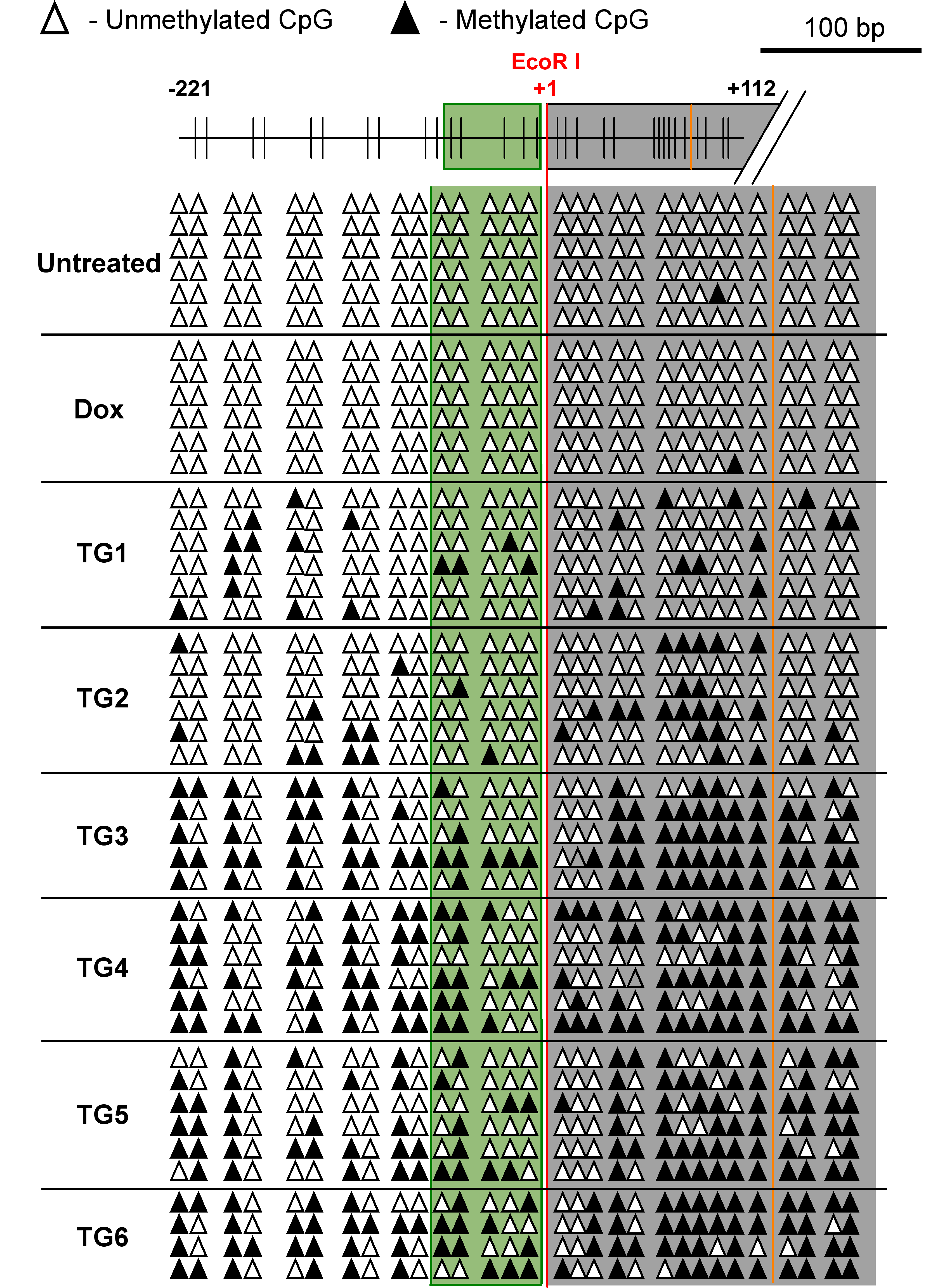

Supplement: Figure S2 — PTRE-HPRT inactivation correlates with increased promoter DNA methylation. Expanded allelic methylation patterns for parental HP14 cells expressing high levels of HPRT (Untreated), reduced levels of HPRT after a 1 week Dox treatment (Dox), and HP14-derived TG-resistant clones (TG1–TG6). Bisulfite sequencing identified methylated (closed triangles) and unmethylated (open triangles) CpG sites within individual alleles. Schematic of the promoter shows approximate positioning of CpG sites (vertical bars) within the minimal CMV promoter (green shaded box) and the 5′ region (∼112 bp) of the HPRT cDNA sequence (grey shaded box). The start of the HPRT cDNA sequence, EcoR I restriction site (red vertical bar), has been designated base position +1. The HPRT start codon is marked by the orange vertical bar. (3.99 MB TIF) [file pone.0004832.s002.tif]

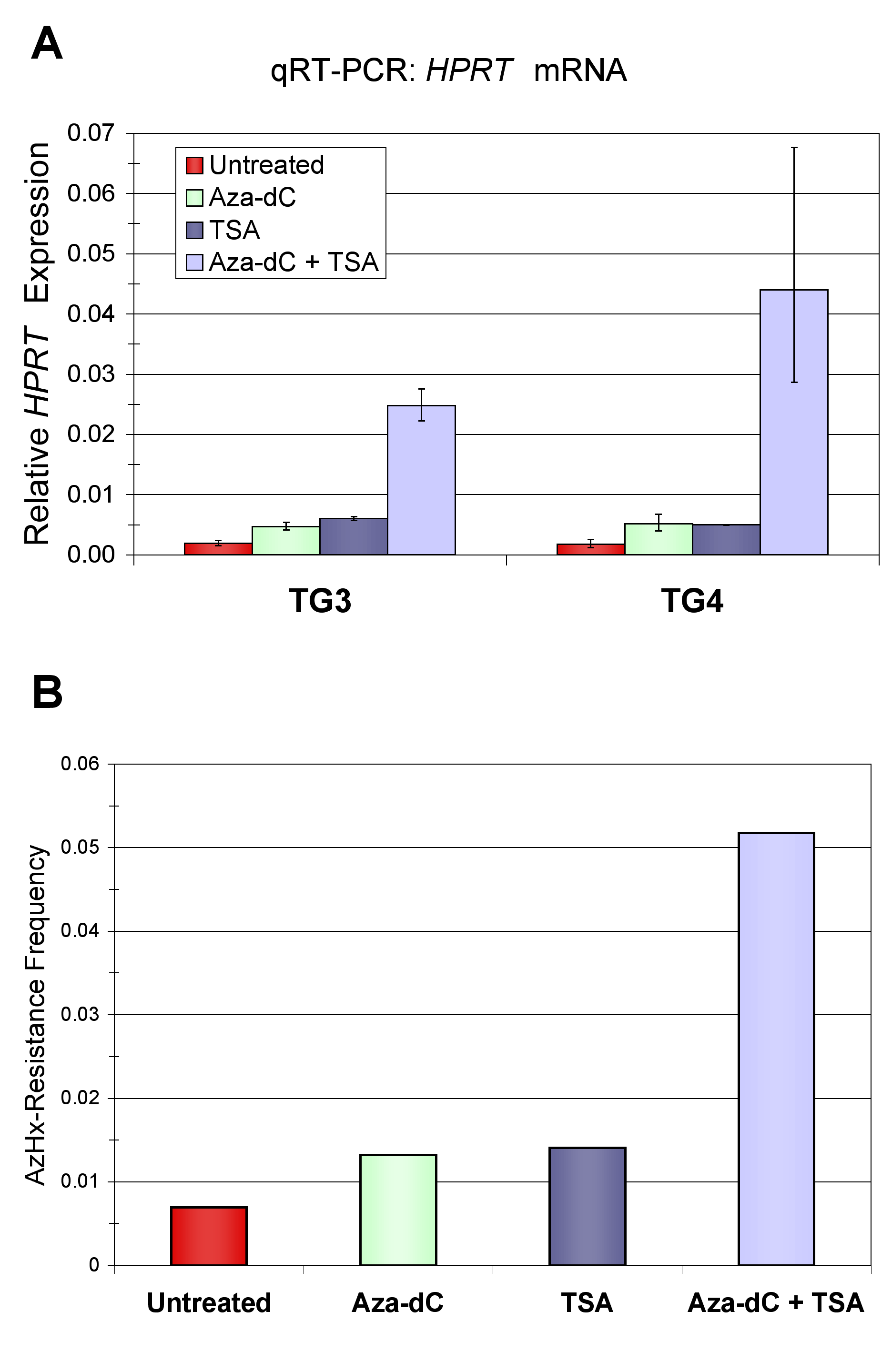

Supplement: Figure S3 — Silenced PTRE-HPRT alleles are reactivated by inhibiting histone deacetylation or DNA methylation. (A) Inhibition of DNA methylation and histone deacetylation increased HPRT mRNA levels. HP14-derived TG-resistant cell lines (TG3 and TG4) were treated with 300 nM 5-aza-dC (Aza-dC), inhibiting histone deacetylation with 100 nM trichostatin A (TSA), or a combination of the 300 nM 5-aza-dC and 100 nM TSA treatments (Aza-dC+TSA). Cells were treated with inhibitors overnight (∼16 hours), and RNA was harvested 24 hours later. The units shown along the Y-axis are relative to those measured in the untreated parental HP14 cells (see Figure 3). HPRT expression was measured by qRT-PCR and normalized to Gapdh expression levels. Each bar represents the average of duplicate reactions with error bars indicating minimum and maximum fold change. (B) TG-resistant cell lines were capable of stably reactivating PTRE-HPRT expression as shown by reversion assay. Cells were plated with azaserine/hypoxanthine (AzHx) selection, which requires HPRT enzyme activity for cell survival, to isolate and measure the number of cells that stably reactivated HPRT expression. Before plating and selection, cells were treated overnight with 300 nM 5-aza-dC (Aza-dC), 100 nM TSA (TSA), both 300 nM 5-aza-dC and 100 nM TSA (Aza-dC+TSA), or vehicle control (Untreated) and allowed to recover for 24 hours. Frequencies represent the fraction of cell colonies surviving after two weeks of continuous AzHx selection. (0.26 MB TIF) [file pone.0004832.s003.tif]

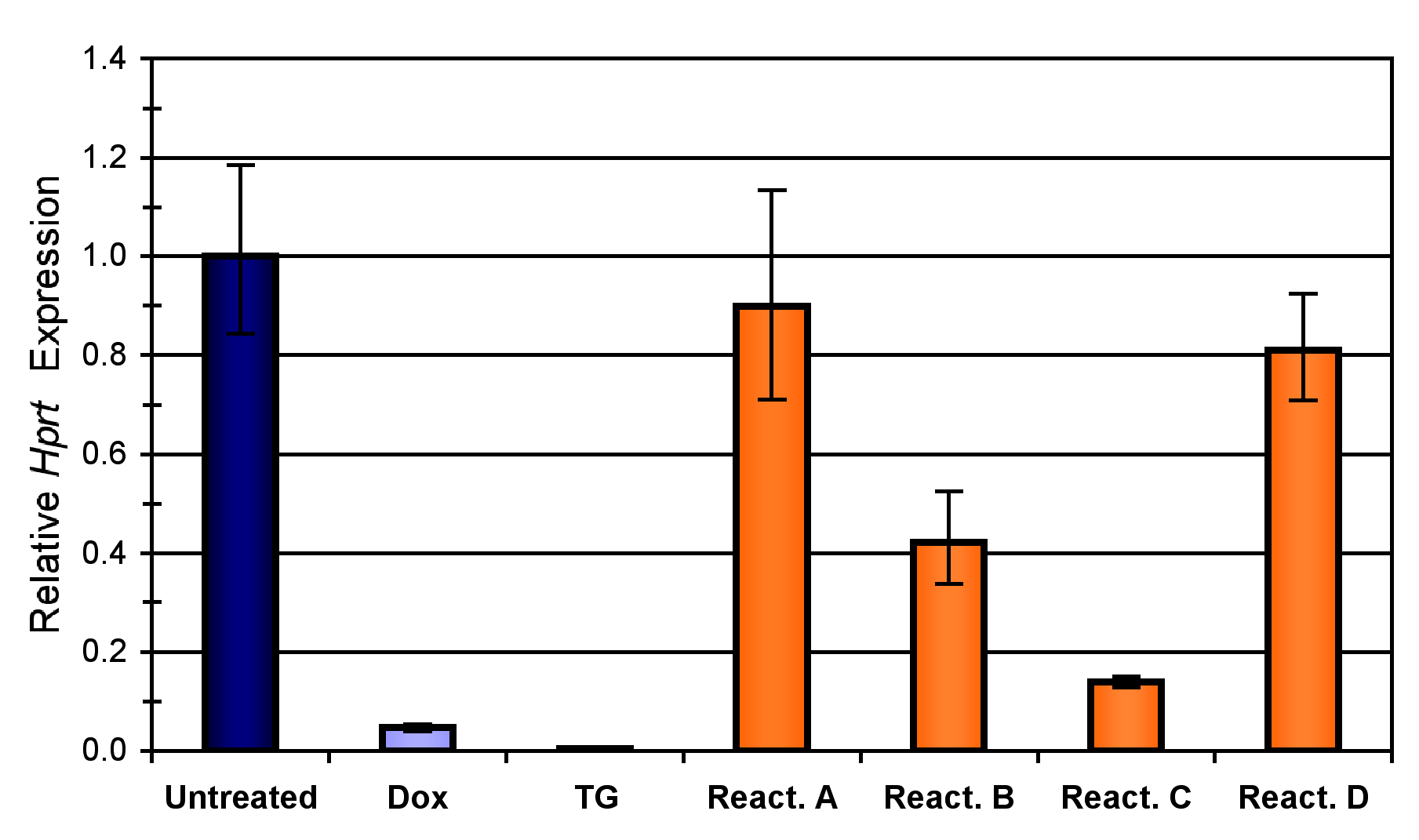

Supplement: Figure S4 — Reactivant cell lines have increased HPRT expression levels. HPRT mRNA levels were measured in reactivant cell lines (Reactivants A, B, C, and D) and displayed relative to the expression level in the initial HP13 parental cell line (Untreated). Also shown for comparison are HPRT expression levels in the TG-resistant cell line before reactivation (TG), and the parental line after treatment with Dox for one week (Dox). HPRT expression was measured by qRT-PCR and normalized to Gapdh expression levels. Each bar represents the average of duplicate reactions with error bars indicating minimum and maximum fold change. (0.18 MB TIF) [file pone.0004832.s004.tif]
